# Cholera

**Published:** 1873-08

**Authors:** 


CHOLRRA has been rampant in many cities and towns of the country. We have had no experience with the "dread monster," and hope never to have. Atlanta seems to be cholera-proof. It has swung corners around her, carrying dismay and death in its mad dance, but not a case—except imported—has occurred. In every epidemic in this country (except that of 1832, we believe, before Atlanta existed), cases of cholera have been imported, and have died in the city, but not a single case has originated from them. Atlanta air is much too healthy for the medical man to grow rich in, and the healing virtues of her numerous mineral springs threaten to "stamp out" all the old chronics in her midst. If you wish to live long and enjoy life—provided you will "throw physic to the dogs"—come to Atlanta, breathe her pure air, and quaff her cool, sparkling mineral water. If well, it is the place—if sick, there is no such place to get well in.

